# Analysis of the Asymmetrical Rolling of Ultra-Thin Strips Considering Elastic Deformation of the Strips

**DOI:** 10.3390/ma17102467

**Published:** 2024-05-20

**Authors:** Qilin Zhao, Xianlei Hu, Xianghua Liu

**Affiliations:** 1 School of Materials Science and Engineering, Northeastern University, Shenyang 110819, China; zhaoql73@163.com; 2 State Key Laboratory of Rolling and Automation, Northeastern University, Shenyang 110819, China

**Keywords:** asymmetrical rolling, ultra-thin strip, elastic deformation of the strip, elastic recovery zone, mathematic model, slab method

## Abstract

In normal cold rolling, the elastic deformation of the strip is typically ignored because of the dominant plastic deformation. However, this neglect may introduce additional errors when the strip is very thin. The aim of this study is to investigate the characteristics of the deformation region and thickness reduction in the asymmetrical rolling of ultra-thin strips. Mathematical models were developed based on the slab method, with consideration of the elastic deformation of the strips, and employed in the simulation calculation. The percentage of the three zones and the thickness reduction were analyzed using the simulation results. An increase in the speed ratio results in an increase in the reduction ratio, which is influenced by parameters, such as front tension, back tension, friction coefficient, and entry thickness. The elastic deformation of the strip reduces the tension and the roll pressure and causes the reduction ratio to decrease. The findings and conclusions of this study may be helpful to the mill operating in the asymmetrical rolling process of ultra-thin strips.

## 1. Introduction

In recent years, the boom in consumer electronics, new energy vehicles, and drones has led to increased demand for ultra-thin strips. The products and the production of ultra-thin strips have attracted more and more attention. The advantages of low cost, flexibility, simplicity, lower force requirements, and excellent thinning capacity have attracted increasing interest in asymmetrical rolling.

Researchers have developed many mathematical models to study the process parameters and deformation zone characteristics in asymmetrical rolling.

By using the slab method, Gao et al. [1] built a model to study the effects of the friction coefficient ratio on shear deformation, rolling pressure, and torque. In their models, Salimi [2] and Zhang [3] took shear stress into account. Razani et al. [4] established a new model to calculate the roll force considering the rate-dependent condition of yield stress. Huang and his coworkers [5] built analytical models using the slab method to calculate the mechanical parameters in asymmetrical cold strip rolling, and the finite element method was employed in their further work [6]. Sun et al. [7] classified the deformation zone configuration into six types and built models to calculate the mechanical parameters. In their model, Tian et al. [8] considered the contact arc as a parabola.

The minimum achievable thickness of a mill has always been a limitation in the production of ultra-thin strips. Stone [9,10] proposed the classic formula for calculating the minimum achievable thickness. According to Stone’s formula, the minimum achievable thickness is about 1/1500~1/2000 of the working roll diameter for symmetrical rolling. Compared with symmetrical rolling, the thinning capacity of asymmetrical rolling is excellent, which has attracted the interest of researchers. Zhu et al. [11] obtained a 0.005 mm copper strip by asymmetrical rolling in a four-high mill with a 90 mm work roll, *D*/*h* up to 18,000, and proposed the “elastic plug” theory. Tzou and Huang [12,13] built two analytical models using the slab method to calculate the minimum reliable thickness in PV cold rolling taking different friction conditions into account. Tang et al. [14] found that the minimum thickness of asymmetrical rolling only exists within a specific range of the percentage of the cross-shear zone. Finite element and theoretical analytical models were combined by Feng et al. [15] to investigate the minimum thickness of single-roll-driven asymmetric rolling. Based on the analysis of the relationship between the deformation zone configuration and rolling parameters, Wang et al. [16] suggested that the minimum thickness can be reached at the disappearance of the forward-slip zone.

Over the past two decades, research on asymmetric rolling has increasingly focused on its application.

Asymmetrical rolling is used to produce composite plates and multilayer materials. The effects of process parameters on the asymmetric rolling of unbonded clad sheets were investigated by Afrouz and Parvizi [17]. Qwamizadeh et al. [18] proposed a theoretical model based on the slab method to analyze the asymmetrical rolling of bonded two-layer sheets and estimated the curvature of the sheet at the exit of the roll gap. Zhi et al. [19] studied the effects of different thickness ratios of component metals on the microstructure, interface structure, and mechanical properties of composite plates. A model was built by Jiang et al. [20] to predict the plate curvature of a double-layer clad plate using the flow function method and the upper bound method. Guo et al. [21] established a simulation model of TC4/304 clad plate rolling by the finite element method and studied the effect of rolling temperature on the interfacial bonding of clad plates.

Since asymmetrical rolling can cause significant microstructure changes, it is increasingly employed to improve grain size refinement, resulting in enhanced mechanical properties and formability. Nam and his coworkers [22] improved the R-value of an AA1050 sheet to 2.13 times its original value by heat treatment after asymmetrical rolling. Amegadzie et al. [23] conducted an asymmetrical rolling experiment to investigate the influences of rolling parameters on the mechanical properties of AA6061. Li et al. [24] found the strengthening mechanism of medium-carbon low-alloy during asymmetric rolling. Xiao et al. [25] employed asymmetrical rolling to refine grains in order to investigate the mechanical properties of pure titanium foils. By comparing the microstructure, texture, and mechanical properties of TZ61 and AZ61, Majchrowicz et al. [26]. found that the plasticity of annealed TZ61 and AZ61 sheets increased because of the reduced grain size and weakened basal texture caused by the asymmetric rolling process. Xu et al. [27] introduced a new asymmetric rolling technology in pure copper plate rolling and achieved a reduction in grain size of 5.77%, 17.3%, and 21.7% on the upper surface, center, and lower surface, respectively.

Although numerous research experiments, theoretical models, and applications of asymmetric rolling have been carried out, few have focused on the mathematical model for thin strips, especially for the ultra-thin strips that must take the elastic deformation of the strip into account. Therefore, further study of theoretical models is necessary. In this paper, analytical models for asymmetrical rolling of ultra-thin strips are developed based on the slab method, considering the elastic deformation of the strips. The proposed models are used to calculate the percentages of three zones, reduction, roll pressure, and roll force in the asymmetrical rolling of ultra-thin strips. The effects of elastic deformation of the strip on the percentages of the three zones and the reduction ratio are analyzed. The calculated values are in good agreement with the experimental results.

## 2. Mathematical Models

**Figure 1:**
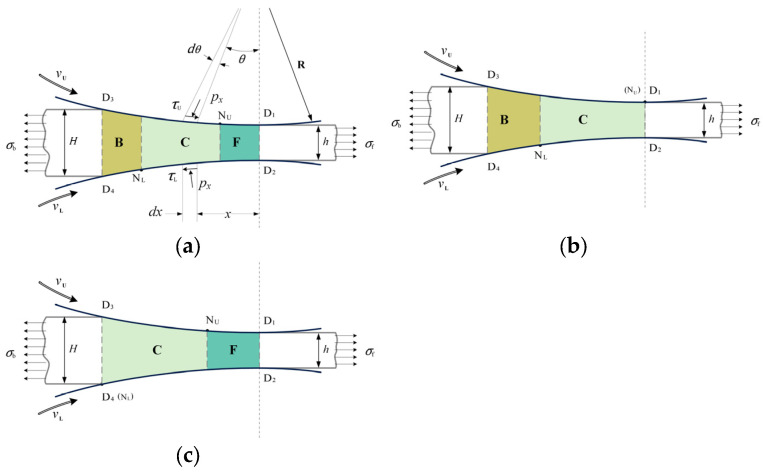
Schematic of deformation region states in asymmetrical strip rolling: (**a**) B+C+F, (**b**) B+C, and (**c**) C+F.

The typical deformation region in asymmetrical rolling consists of the backward-slip zone (B), the cross-shear zone (C), and the forward-slip zone (F), as illustrated in Figure 1a.

The percentages of the three zones vary with the position of the neutral point. When the upper neutral point (N_U_) reaches the exit of the deformation region, the percentage of the forward-slip zone becomes zero (Figure 1b). Conversely, when the lower neutral point (N_L_) reaches the entry of the deformation region, the backward-slip zone disappears (Figure 1c). The states of the deformation region, as illustrated in Figure 1a, 1b and 1c, are designated as B+C+F, B+C, and C+F, respectively.

### 2.1. Basic Assumptions

The following assumptions are used to derive the models for the asymmetric rolling of ultra-thin strips:

(1) Strips are considered rigid-plastic materials in the deformation region and elastic materials outside.
(2) The upper and lower rolls are rigid bodies with identical diameters.
(3) The friction coefficients may be different on the upper and lower surfaces of the strip, and they are assumed to remain constant within the deformation region.
(4) The plane strain is assumed.
(5) The normal stress and the horizontal stress are regarded as principal stresses, and it is assumed that they are uniformly distributed throughout the slab.
(6) A string is employed to represent the contact arc.

### 2.2. Roll Pressure

In the typical deformation region state of asymmetrical strip rolling illustrated in Figure 1a, *R* is the roll radius; $v_{U}$ and $v_{L}$ are peripheral velocities of the upper and lower rolls, and $v_{U}\geq v_{L}$; *θ* is the rolling angle; *H* is the entry thickness; *h* is the exit thicknesses; and *x* is the distance to the exit of the deformation region.

**Figure 2:**
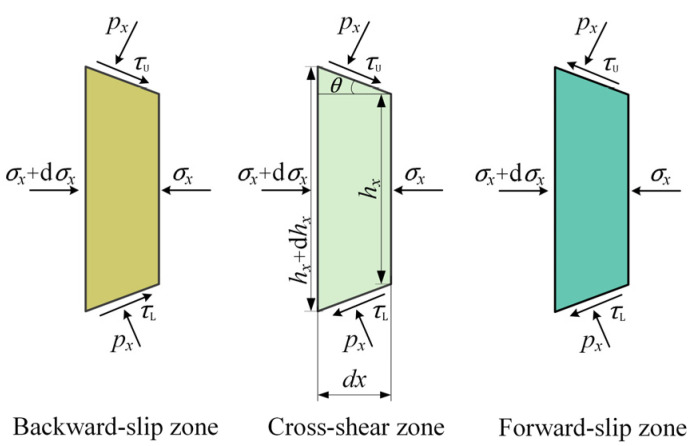
Stress sates of slabs taken from different deformation zones.

Slabs were taken from the different deformation zones shown in Figure 1a. The stress state of these slabs is illustrated in Figure 2, where $p_{x}$ and $\sigma_{x}$ are the normal pressure and the horizontal stress, respectively; $\tau_{U}$ and $\tau_{L}$ are upper and lower friction stresses; and $h_{x}$ is the strip thickness at location *x*.

Based on the stresses shown in Figure 2, the equilibrium of forces in the horizontal direction for the backward-slip, cross-shear, and forward-slip zones can be obtained as follows:

(1) $$\left\{\begin{array}{c} (\sigma_{x}+d\sigma_{x})(h_{x}+dh_{x})-\sigma_{x}h_{x}-2p_{x}Rd\theta sin\theta +(\tau_{U}+\tau_{L})Rd\theta \cos\theta =0\\ (\sigma_{x}+d\sigma_{x})(h_{x}+dh_{x})-\sigma_{x}h_{x}-2p_{x}Rd\theta sin\theta +(\tau_{U}-\tau_{L})Rd\theta \cos\theta =0\\ (\sigma_{x}+d\sigma_{x})(h_{x}+dh_{x})-\sigma_{x}h_{x}-2p_{x}Rd\theta sin\theta -(\tau_{U}+\tau_{L})Rd\theta \cos\theta =0 \end{array}\right.$$

The friction stresses $\tau_{U}$ and $\tau_{L}$ can be expressed as:

(2) $$\left\{\begin{array}{l} \tau_{U}=\mu_{U}p_{x}\\ \tau_{L}=\mu_{U}p_{x} \end{array}\right.$$

Substituting Equation (2) into Equation (1) and ignoring higher-order infinitesimals, Equation (1) is simplified to:

(3) $$\left\{\begin{array}{c} -h_{x}d\sigma_{x}-\sigma_{x}dh_{x}+2p_{x}\tan\theta dx+(\mu_{U}+\mu_{L})p_{x}dx=0\\ -h_{x}d\sigma_{x}-\sigma_{x}dh_{x}+2p_{x}\tan\theta dx-(\mu_{U}-\mu_{L})p_{x}dx=0\\ -h_{x}d\sigma_{x}-\sigma_{x}dh_{x}+2p_{x}\tan\theta dx-(\mu_{U}+\mu_{L})p_{x}dx=0 \end{array}\right.$$

Since the thickness reduction in ultra-thin strip rolling is minimal, $dh_{x}\to 0$ and tanθ∼0. Therefore, Equation (3) becomes:

(4) $$\left\{\begin{array}{c} \bar{h}d\sigma_{x}-(\mu_{U}+\mu_{L})p_{x}dx=0\\ \bar{h}d\sigma_{x}+(\mu_{U}-\mu_{L})p_{x}dx=0\\ \bar{h}d\sigma_{x}+(\mu_{U}+\mu_{L})p_{x}dx=0 \end{array}\right.$$

where $\bar{h}=\frac{H+h}{2}$ is the mean thickness in the plastic deformation region.

The von Mises yield criterion in plane strain is [28]:

(5) $$p_{x}-\sigma_{x}=K$$

where *K* is the plane deformation resistance of the strip.

Substituting Equation (5) into Equation (4) gives the following:

(6) $$\left\{\begin{array}{l} dp_{x}=-\delta_{1}\;p_{x}\;dx\\ dp_{x}=\delta_{2}\;p_{x}\;dx\\ dp_{x}=\delta_{1}\;p_{x}\;dx \end{array}\right.$$

where $\delta_{1}=\frac{\mu_{U}+\mu_{L}}{\bar{h}}$ and $\delta_{2}=\frac{\mu_{L}-\mu_{U}}{\bar{h}}$.

The solutions of Equation (6) are obtained by integration as follows:

(7) $$\left\{\begin{array}{c} \ln p_{x}\\ \ln p_{x}\\ \ln p_{x} \end{array}\begin{array}{c} =\\ =\\ = \end{array}\begin{array}{c} -\delta_{1}\;x+C_{B}\\ \delta_{2}\;x+C_{C}\\ \delta_{1}\;x+C_{F} \end{array}\right.$$

where $C_{B}$, $C_{C}$, and $C_{F}$ are integral constants for the backward-slip zone, the cross-shear zone, and the forward-slip zone, respectively.

At the entry of the deformation region, the boundary conditions are as follows:

(8) $$\left\{\begin{array}{l} x=l\\ p_{x}=K-\sigma_{b} \end{array}\right.$$

and at the exit of the deformation region, the boundary conditions are:

(9) $$\left\{\begin{array}{l} x=0\\ p_{x}=K-\sigma_{f} \end{array}\right.$$

where $\sigma_{f}$ is the front tension and $\sigma_{b}$ is the back tension. On the interface between the forward-slip zone and the cross-shear zone, the boundary conditions are:

(10) $$\left\{\begin{array}{l} x=l_{F}\\ p_{C}=p_{F} \end{array}\right.$$

where $l_{F}$ is the length of the forward-slip zone.

By substituting Equations (8) and (9) into Equation (7), $C_{B}$ and $C_{F}$ can be obtained. Then, $C_{C}$ can be determined from Equation (7) and $p_{F}$. The roll pressures for the deformation region state B+C+F (Figure 1a) can be expressed as follows:

(11) $$\left\{\begin{array}{l} p_{F}=(K-\sigma_{f})e^{\;\delta_{1}\;x}\\ p_{C}=(K-\sigma_{f})e^{(\delta_{1}-\delta_{2})\;l_{F}\;}e^{\;\delta_{2}\;x}\\ p_{B}=(K-\sigma_{b})e^{\;\delta_{1}\;(l-x)} \end{array}\right.$$

Similarly, the roll pressures for the deformation region state B+C (Figure 1b) can be expressed as follows:

(12) $$\left\{\begin{array}{l} p_{C}=(K-\sigma_{f})e^{\;\delta_{2}\;x}\\ p_{B}=(K-\sigma_{b})e^{\;\delta_{1}\;(l-x)} \end{array}\right.$$

and for the deformation region state C+F (Figure 1c),

(13) $$\left\{\begin{array}{l} p_{F}=(K-\sigma_{f})e^{\;\delta_{1}\;x}\\ p_{C}=(K-\sigma_{b})e^{-\delta_{2}\;(l-x)} \end{array}\right.$$

are obtained.

### 2.3. Percentages of the Three Zones

**Figure 3:**
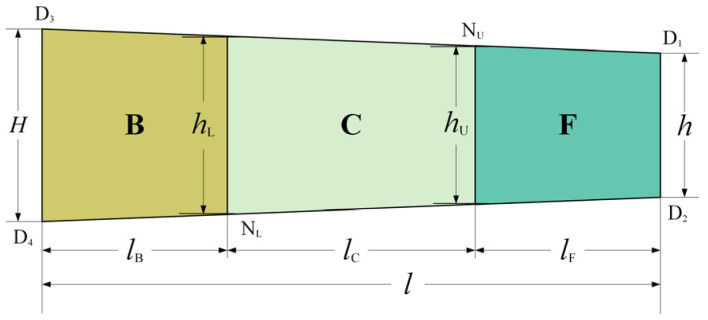
Schematic of the simplified geometry of the plastic deformation region in the asymmetrical rolling of strips.

Figure 3 shows a schematic of the simplified geometry of the plastic deformation region in the asymmetrical rolling of strips. The projected contact lengths of the backward-slip zone, the cross-shear zone, and the forward-slip zone are denoted by $l_{B}$, $l_{C}$, and $l_{F}$, respectively. The percentages of three zones are expressed as:

(14) $$\left\{\begin{array}{l} q_{B}=\frac{l_{B}}{l}\\ q_{C}=\frac{l_{C}}{l}\\ q_{F}=\frac{l_{F}}{l} \end{array}\right.$$

where *l* is the contact length of the plastic deformation region.

The mass flow relationship in the plastic deformation region is:

(15) $$v_{H}H=v_{L}h_{L}=v_{U}h_{U}=v_{h}h$$

where $v_{H}$ and $v_{h}$ are the entry and exit strip velocities and $h_{L}$ and $h_{U}$ are strip thickness at the upper and lower neutral points, respectively. Referring to the geometry shown in Figure 3, $h_{L}$ and $h_{U}$ can be expressed as follows:

(16) $$\left\{\begin{array}{l} h_{U}=h+q_{F}∆h\\ h_{L}=H-q_{B}∆h \end{array}\right.$$

Substitution of Equation (16) into Equation (15) yields:

(17) $$v_{H}H=v_{L}(H-q_{B}∆h)=iv_{L}(h+q_{F}∆h)=v_{h}h$$

where $i=v_{U}/v_{L}$ is the speed ratio.

Substituting Equations (11) and (14) into Equation (10) and combining with Equation (17) gives the three zone percentages of the deformation region state B+C+F:

(18) $$\left\{\begin{array}{l} q_{F}=\frac{1}{1+\lambda i}\left[\frac{\zeta }{\mu_{U}}-\frac{h}{∆h}\lambda (i-1)+\frac{1}{2}(\lambda +1)\right]\\ q_{C}=\frac{1}{1+\lambda \;i}\left[-\frac{\zeta }{\mu_{U}}i-\frac{H}{∆h}(i-1)+\frac{1}{2}(\lambda +1)i\right]\\ q_{B}=\frac{1}{1+\lambda \;i}\left[\frac{\zeta }{\mu_{U}}(i-1)+\frac{h}{∆h}(\lambda +1)(i-1)+\frac{1}{2}(\lambda +1)(i-1)\right] \end{array}\right.$$

where $\zeta =\frac{\bar{h}}{2\;l}\ln\left(\frac{K-\sigma_{b}}{K-\sigma_{f}}\right)$, $\lambda =\frac{\mu_{L}}{\mu_{U}}$.

When the deformation region state is B+C, $q_{F}$ is zero. The roll pressure of the backward-slip zone and that of the cross-shear zone are equal at the interface between them:

(19) $$p_{B}=p_{C}$$

By substituting Equations (12) and (14) into Equation (19), $q_{B}$ and $q_{C}$ are obtained:

(20) $$\left\{\begin{array}{l} q_{C}=\frac{1}{2}(1+\frac{1}{\lambda })-\frac{\zeta }{\mu_{L}}\\ q_{B}=\frac{1}{2}(1-\frac{1}{\lambda })+\frac{\zeta }{\mu_{L}} \end{array}\right.$$

When the deformation region state is C+F, $q_{B}$ is zero. The percentages of C and F are expressed as follows:

(21) $$\left\{\begin{array}{l} q_{F}=\frac{1}{2}(1-\lambda )+\frac{\zeta }{\mu_{U}}\\ q_{C}=\frac{1}{2}(1+\lambda )-\frac{\zeta }{\mu_{U}} \end{array}\right.$$

### 2.4. Critical Speed Ratio

When the state of the deformation region is B+C+F, $q_{F}$ and $q_{B}$ decrease with the increase in the speed ratio. If $q_{F}$ reaches zero first, the deformation region state changes to B+C. On the contrary, if $q_{B}$ reaches zero first, the deformation region state becomes C+F. The speed ratios, at which the forward-slip zone or the backward-slip zone disappears, are defined as critical speed ratios, denoted by $i_{\mathrm{cF}}$ and $i_{\mathrm{cB}}$, respectively.

Setting $q_{F}=0$, $i_{\mathrm{cF}}$ is determined from Equation (18):

(22) $$i_{c\;F}=1+\frac{∆h}{h\;\mu_{L}}\left[\zeta +\frac{1}{2}(\mu_{U}+\mu_{L})\right]$$

Similarly, by setting $q_{B}=0$, $i_{c\;B}$ is obtained:

(23) $$i_{c\;B}=\frac{2\mu_{U}(∆h+h)}{2\mu_{U}\;h+∆h(\;2\zeta +\mu_{U}-\mu_{L})}$$

### 2.5. Roll Force

The normal roll pressure is integrated over the contact arc to determine the roll force per unit width:

(24) $$P_{\left(B+C+F\right)}=\int_{0}^{l_{F}}p_{F(B+C+F)}dx+\int_{l_{F}}^{l_{F}+l_{C}}p_{C(B+C+F)}dx+\int_{l-l_{B}}^{l}p_{B(B+C+F)}dx$$

(25) $$P_{\left(B+C\right)}=\int_{0}^{l_{C}}p_{C(B+C)}dx++\int_{l-l_{B}}^{l}p_{B(B+C)}dx$$

(26) $$P_{\left(C+F\right)}=\int_{0}^{l_{F}}p_{F(C+F)}dx++\int_{l-l_{C}}^{l}p_{C(C+F)}dx$$

Roll pressures are substituted into Equations (24)–(26). Integration gives the roll force per unit width for different deformation region states as follows:

(27) $$P_{\left(B+C+F\right)}=(K-\sigma_{b})\frac{\left(e^{\delta_{1}\;l_{B}}-1\right)}{\delta_{1}}+(K-\sigma_{f})\frac{e^{\delta_{1}\;l_{F}}(e^{\delta_{2}\;l_{C}}-1)}{\delta_{2}}+(K-\sigma_{f})\frac{\left(e^{\delta_{1}\;l_{F}}-1\right)}{\delta_{1}}$$

(28) $$P_{\left(B+C\right)}=(K-\sigma_{b})\frac{\left(e^{\delta_{1}\;l_{B}}-1\right)}{\delta_{1}}+(K-\sigma_{f})\frac{\left(e^{\delta_{2}\;l_{C}}-1\right)}{\delta_{2}}$$

(29) $$\begin{array}{cc} P_{\left(C+F\right)} & = \end{array}(K-\sigma_{f})\frac{\left(e^{\delta_{1}\;l_{F}}-1\right)}{\delta_{1}}-(K-\sigma_{b})\frac{\left(e^{-\delta_{2}\;l_{C}}-1\right)}{\delta_{2}}$$

### 2.6. Roll Torque

By integrating the moment of friction force around the roll axis over the contact arc, the roll torques per unit width are expressed as follows:

(30) $$T_{U}=\int_{\gamma_{2}}^{\alpha }Rp_{B}\mu_{U}Rd\theta +\int_{\gamma_{1}}^{\gamma_{2}}Rp_{C}\mu_{U}Rd\theta -\int_{0}^{\gamma_{1}}Rp_{F}\mu_{U}Rd\theta$$

(31) $$T_{L}=\int_{\gamma_{2}}^{\alpha }Rp_{B}\mu_{L}Rd\theta -\int_{\gamma_{1}}^{\gamma_{2}}Rp_{C}\mu_{L}Rd\theta -\int_{0}^{\gamma_{1}}Rp_{F}\mu_{L}Rd\theta$$

where $\gamma_{1}$ is the upper neutral angle, $\gamma_{2}$ is the lower neutral angle, α is the bite angle.

Substituting Equation (11) into Equations (30) and (31), the roll torques per unit width for the deformation region state B+C+F are calculated:

(32) $$T_{U(B+C+F)}=R\mu_{U}\left[(K-\sigma_{b})\frac{\left(e^{\delta_{1}\;l_{B}}-1\right)}{\delta_{1}}+(K-\sigma_{f})\frac{e^{\delta_{1}\;l_{F}}(e^{\delta_{2}\;l_{C}}-1)}{\delta_{2}}-(K-\sigma_{f})\frac{\left(e^{\delta_{1}\;l_{F}}-1\right)}{\delta_{1}}\right]$$

(33) $$T_{L(B+C+F)}=R\mu_{L}\left[(K-\sigma_{b})\frac{\left(e^{\delta_{1}\;l_{B}}-1\right)}{\delta_{1}}-(K-\sigma_{f})\frac{e^{\delta_{1}\;l_{F}}(e^{\delta_{2}\;l_{C}}-1)}{\delta_{2}}-(K-\sigma_{f})\frac{\left(e^{\delta_{1}\;l_{F}}-1\right)}{\delta_{1}}\right]$$

Similarly, the roll torques per unit width for the state B+C can be expressed as follows:

(34) $$T_{U(B+C)}=R\mu_{U}\left[(K-\sigma_{b})\frac{\left(e^{\delta_{1}\;l_{B}}-1\right)}{\delta_{1}}+(K-\sigma_{f})\frac{\left(e^{\delta_{2}\;l_{C}}-1\right)}{\delta_{2}}\right]$$

(35) $$T_{L(B+C)}=R\mu_{L}\left[(K-\sigma_{b})\frac{\left(e^{\delta_{1}\;l_{B}}-1\right)}{\delta_{1}}-(K-\sigma_{f})\frac{\left(e^{\delta_{2}\;l_{C}}-1\right)}{\delta_{2}}\right]$$

The roll torques per unit width for the state C+F can be expressed as follows:

(36) $$T_{U(C+F)}=R\mu_{U}\left[-(K-\sigma_{f})\frac{\left(e^{\delta_{1}\;l_{F}}-1\right)}{\delta_{1}}-(K-\sigma_{b})\frac{\left(e^{-\delta_{2}\;l_{C}}-1\right)}{\delta_{2}}\right]$$

(37) $$T_{L(C+F)}=R\mu_{L}\left[-(K-\sigma_{f})\frac{\left(e^{\delta_{1}\;l_{F}}-1\right)}{\delta_{1}}+(K-\sigma_{b})\frac{\left(e^{-\delta_{2}\;l_{C}}-1\right)}{\delta_{2}}\right]$$

The required total roll torque is the sum of the upper roll torque and the lower roll torque.

### 2.7. Pressure Distribution in Elastic Zones

**Figure 4:**
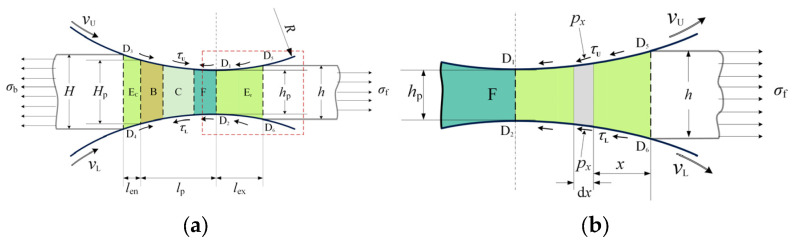
Schematics of the (**a**) contact region with the elastic compression zone and the elastic recovery zone and (**b**) the elastic recovery zone.

The entire contact zone in strip rolling comprises the elastic compression zone (Ec), the plastic deformation region (B, C, F) and the elastic recovery zone (Er), as illustrated in Figure 4a. Figure 4b illustrates a more detailed schematic of the elastic recovery zone. D_1_D_2_ represents the exit plane of the plastic deformation region. The strip recovers elastically to the exit thickness *h* at D_5_D_6_.

Since the thickness of the strip is very thin, the normal pressure is regarded as the principal stress in the vertical direction. In the horizontal direction, the front tension at location *x* is expressed as:

(38) $$\sigma_{x}=\frac{h}{h_{x}}\sigma_{f}-\frac{\left(\mu_{U}+\mu_{L}\right)}{h_{x}}\int_{0}^{x}p_{x}dx$$

where $h_{x}$ is thickness of the strip at *x* and $p_{x}$ is the normal pressure at *x*.

The elastic deformation in the vertical direction can be expressed by employing Hooke’s law:

(39) $$\frac{h-h_{x}}{h}=\frac{1-\nu^{2}}{E}\left[p_{x}+\frac{\nu }{1-\nu }\left(\frac{h}{h_{x}}\sigma_{f}-\frac{\left(\mu_{U}+\mu_{L}\right)}{h_{x}}\int_{0}^{x}p_{x}dx\right)\right]$$

The elastic recovery zone is divided into slabs of width ∆x. The thickness and pressure at location *x* are represented by $h_{n}$ and $p_{n}$, respectively. The relationship between thickness and pressure is expressed as:

(40) $$p_{n}=\frac{\frac{E\left(h-h_{n}\right)h_{n}}{h}-\left(1+\nu \right)\nu h\sigma_{f}+\left(\mu_{U}+\mu_{L}\right)\left(1+\nu \right)\nu ∆x\sum_{j=1}^{n-1}p_{j}}{\left(1-\nu^{2}\right)h_{n}-\left(\mu_{U}+\mu_{L}\right)\left(1+\nu \right)\nu ∆x}$$

The initial thickness distribution in the elastic recovery zone is obtained by assuming a circular profile of the roll. Then, the pressure distribution in the elastic recovery zone is calculated, beginning with $h_{0}=h$ and $p_{0}=0$ and ending with $p_{n}-\sigma_{n}=K$, by using Equations (38) and (40). The thickness and pressure distributions are calculated interactively until a convergence is reached.

The thickness and pressure distributions in the elastic compression zone are obtained following the same method.

## 3. Experiments and Simulations

**Figure 5:**
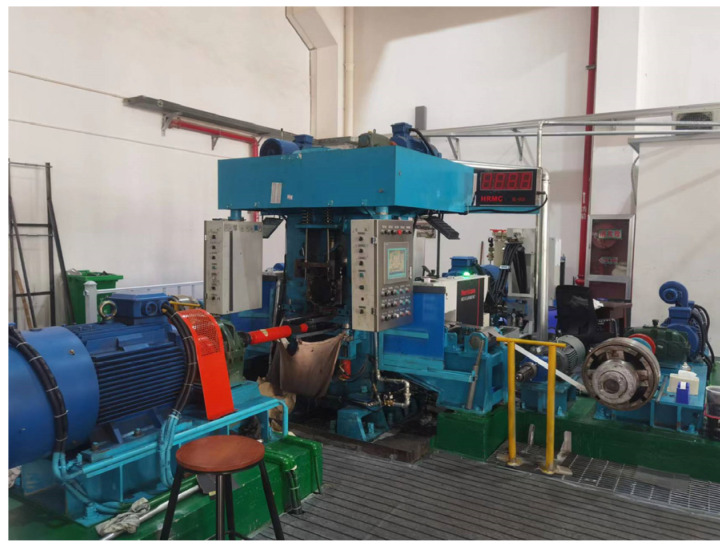
Photo of the experimental mill.

Experimental rolling was carried out on the four-high reversing mill shown in Figure 5. The diameter of the work roll is 90 mm, and the work rolls are driven separately. The maximum allowable roll force is 800 kN. Measured values of roll force, roll torque, roll speed, and strip thickness can be obtained using the equipped apparatus. In the experiment, work rolls with a diameter of 90.0 mm were employed, with the lower (slow) roll speed set at 4.0 m/min. Stainless steel (430) strips, with a thickness of 0.06 mm and 0.07 mm and a width of 120 mm, were rolled with emulsion lubrication.

The proposed models were employed in the simulation of the asymmetrical rolling of ultra-thin strips, from which roll pressure, the percentages of the three zones, roll force, and roll torque are calculated. A peripheral speed of 1.0 m/s was used for the slow roll, and the peripheral speed of the fast roll was determined by the speed ratio. Ten specimens were cut from different locations of the strip and subjected to tensile testing. The 10 tensile test datasets were employed to fit the deformation resistance curve of the 430 stainless steel strip.

(41) $$K=\frac{2}{\sqrt{3}}\left(363.8+319.7{\bar{\varepsilon }}^{0.496}\right)$$

where $\bar{\varepsilon }=1-\left(0.4H+0.6h\right)/H_{0}$ is the average strain and $H_{0}$ is the initial thickness of the strip.

## 4. Results and Discussion

In the following discussion, the speed ratio of 1.0 refers to symmetrical rolling. The legends with “elastic” in the figures represent values that take elastic zones into account.

### 4.1. Analysis of the Three Zone Percentages

**Figure 6:**
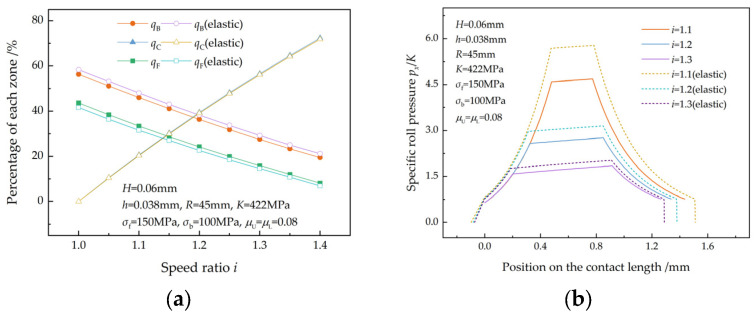
Variations in the (**a**) percentages of the three zones and (**b**) specific roll pressure with the speed ratio in the case of equal reduction.

Figure 6 illustrates the effect of the speed ratio on the percentages of the three zones and specific roll pressures in the case of an equal reduction ratio. As can be seen from Figure 6a, $q_{B}$ and $q_{F}$ decrease as the speed ratio increases, while $q_{C}$ increases with the speed ratio. This trend was also found in the research of Sun et al. [7]. An increase in the speed ratio results in the movements of the upper and lower neutral points towards the exit and entry of the deformation region, respectively. Consequently, there is an increase in $q_{C}$ and a decrease in $q_{B}$ and $q_{F}$. As a result of the elastic deformation of the strip, $q_{B}$ increases, $q_{F}$ decreases, and $q_{C}$ remains unchanged. When there are elastic zones, friction in elastic zones results in a reduction in the front and back tension acting on the plastic deformation region. Since the reduction in the front tension is greater than that in the back tension, both the upper and lower neutral points move towards the exit of the deformation region. As a result, the percentage of the forward-slip zone decreases, and the percentage of the backward-slip zone increases. As the speed ratio increases, the average specific roll pressure decreases because of the increase in $q_{C}$ (Figure 6b). This is in accordance with the findings of Sun et al. [7] and Tian et al. [8]. More thickness reduction is required to achieve the desired exit thickness because of the elastic recovery of the strip, resulting in a higher specific roll pressure.

**Figure 7:**
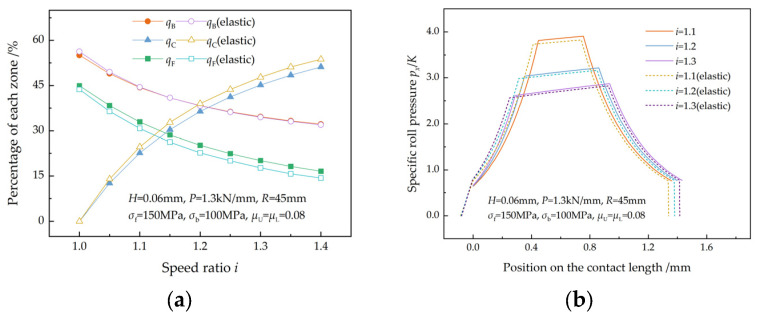
Variations in the (**a**) percentages of the three zones and (**b**) specific roll pressure with the speed ratio in the case of equal roll force.

Figure 7 illustrates the effect of the speed ratio on the percentages of the three zones and roll pressures in the case of equal roll force. As the speed ratio increases, the velocity difference between the upper and lower surfaces increases, causing the neutral points to move towards the entry and exit of the deformation region. Thus, as the speed ratio increases, $q_{B}$ and $q_{F}$ decrease while $q_{C}$ increases, as shown in Figure 7a. Since the roll pressure required to achieve a given reduction can be reduced by increasing $q_{C}$, a greater reduction is obtained at a higher speed ratio, resulting in a longer contact length and a lower specific roll pressure (Figure 7b). Sun et al. [7] reported the same research results.

The elastic recovery zone is larger than the elastic compression zone, which results in a greater reduction in the front tension acting on the plastic deformation region than in the back tension. Consequently, the lower neutral point moves slightly towards the exit of the deformation region, while the upper neutral point moves more obviously in the same direction. The movements of the neutral points lead to an increase in $q_{C}$ and a decrease in $q_{F}$, while almost no change occurred in $q_{B}$ (Figure 7a). In the case of equal roll force, the average specific roll pressure in the plastic deformation region decreased slightly because of the occurrence of specific roll pressure in the elastic zones (Figure 7b).

**Figure 8:**
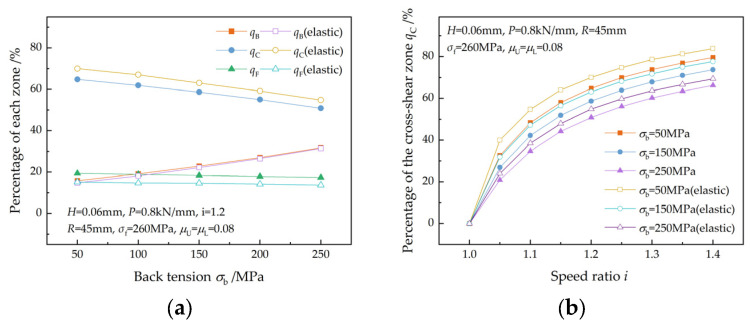
Effects of back tension on (**a**) the percentages of the three zones and (**b**) the percentage of cross-shear zone.

Figure 8 shows the effect of back tension on the percentages of the three zones. At a given speed ratio, $q_{B}$ increases with back tension, while $q_{F}$ and $q_{C}$ decrease with increasing back tension (Figure 8a). The reason for this is that the increase in back tension causes the entry velocity of the strip to slow down, causing both the upper and lower neutral points to move towards the exit of the deformation region. As the speed ratio increases, the velocity difference between the fast roll and the slow roll increases, and the two neutral points move towards the entry and exit of the deformation region, respectively. As a result, $q_{C}$ increases with the speed ratio at the same back tension (Figure 8b). A similar trend in $q_{C}$ was observed in the research of Sun et al. [7] and Wang et al. [16]

The more pronounced reduction in the front tension caused by the friction in the elastic recovery zone results in a more obvious movement of the upper neutral point towards the exit of the deformation region. This leads to an increase in $q_{C}$ and a decrease in $q_{F}$.

**Figure 9:**
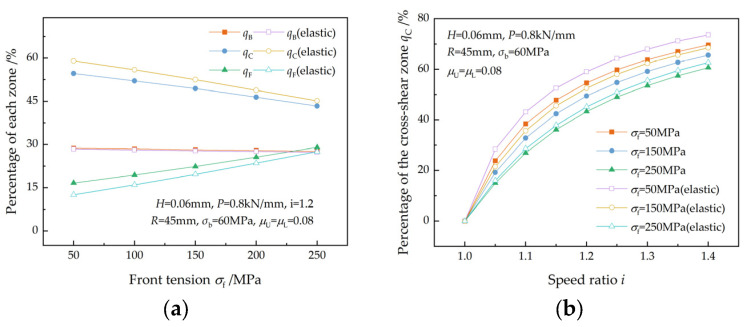
Variations in (**a**) the percentages of the three zones and (**b**) the percentage of the cross-shear zone at different speed ratios with front tension.

The effect of front tension on the percentages of the three zones is illustrated in Figure 9, which is similar to that of back tension. As the front tension increases under the same speed ratio, $q_{B}$ and $q_{C}$ decrease, while $q_{F}$ increases, as shown in Figure 9a. For the same front tension, $q_{C}$ increases with the speed ratio (Figure 9b), which is in agreement with the research results of Sun et al. [7]. A greater change in $q_{C}$ and $q_{F}$ occurs at lower front tension because of the greater reduction in front tension caused by friction in the elastic recovery zone.

**Figure 10:**
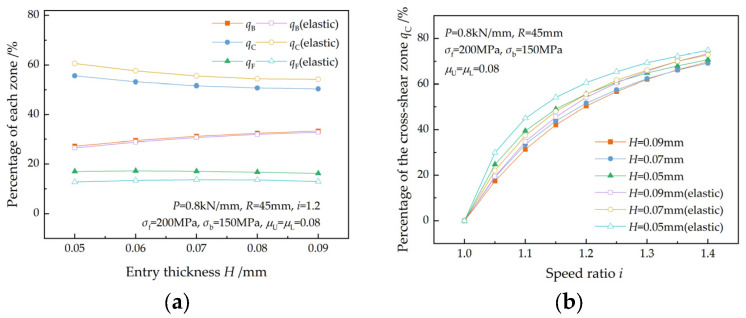
Variations in (**a**) the percentages of the three zones and (**b**) the percentage of the cross-shear zone at different speed ratios with entry thickness.

Figure 10 shows the effect of entry thickness on the percentages of the three zones. As the entry thickness increases, $q_{B}$ and $q_{F}$ increase, while $q_{C}$ decreases (Figure 10a). $q_{C}$ increases with the speed ratio for a given entry thickness and decreases slightly with increasing entry thickness for a given speed ratio (Figure 10a). This trend was also found in reference [7].

The presence of elastic zones leads to an increase in $q_{C}$, a decrease in $q_{F}$, and a negligible decrease in $q_{B}$.

**Figure 11:**
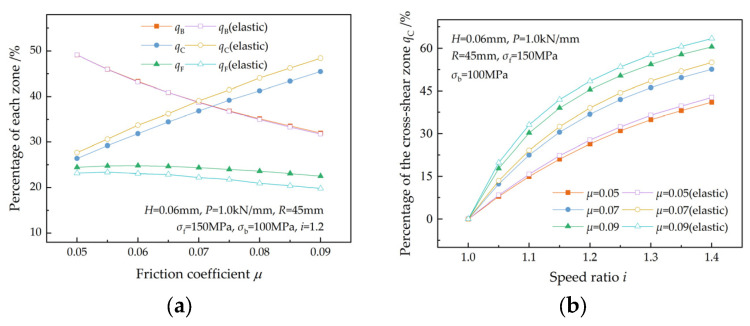
Effect of the friction coefficient on (**a**) the percentage of the three zones and (**b**) the percentage of the cross-shear zone at different speed ratios.

Figure 11 illustrates the variation in the percentages of the three zones with the friction coefficient. For a given speed ratio, an increase in the friction coefficient results in a decrease in $q_{B}$ and $q_{F}$ but an increase in $q_{C}$. Furthermore, $q_{C}$ increases with the speed ratio for a given friction coefficient. The increase in $q_{C}$ with the friction coefficient is more significant at higher speed ratios. This is in accordance with the findings of Sun et al. [7].

The elastic zones cause an increase in $q_{C}$ and a decrease in $q_{F}$, while having minimal effect on $q_{B}$. The changes in $q_{C}$ and $q_{F}$ are more pronounced at higher friction coefficients.

**Figure 12:**
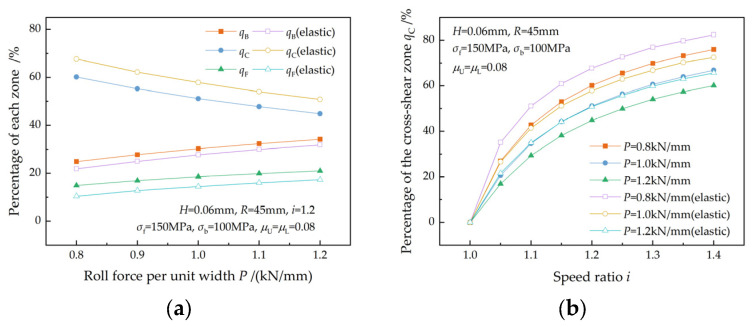
Effects of roll force on (**a**) the percentages of the three zones and (**b**) the percentage of the cross-shear zone at different speed ratios.

Figure 12a illustrates the variations in the percentages of the three zones with roll force at a speed ratio of 1.2. As the roll force increases, $q_{B}$ and $q_{F}$ increase, while $q_{C}$ decreases. Figure 12b shows that $q_{C}$ increases with the speed ratio and increases more rapidly at lower roll force. A similar research result was reported by Sun et al. [7].

When there are elastic zones, $q_{B}$ and $q_{F}$ increase, while $q_{C}$ decreases.

### 4.2. Analysis of Reduction

The increase rate of the reduction ratio at speed ratio *i*, relative to the reduction ratio at speed ratio 1.0, is employed to assess the effect of the speed ratio on enhancing thickness reduction.

(42) $$\varphi_{r}=\frac{r_{i}-r_{0}}{r_{0}}\times 100\%$$

where $\varphi_{r}$ is the increase rate of the reduction ratio and $r_{i}$ and $r_{0}$ are the reduction ratios at speed ratio i and 1.0, respectively. A greater $\varphi_{r}$ means a greater reduction ratio can be achieved.

**Figure 13:**
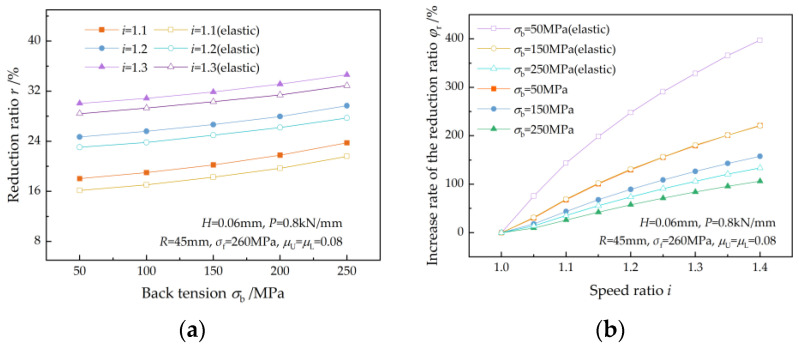
Effects of back tension on (**a**) the reduction ratio and (**b**) the increase rate of the reduction ratio at different speed ratios.

Figure 13 shows the effect of back tension on the reduction ratio. As shown in Figure 13a, the reduction ratio increases with the back tension for a given speed ratio because less roll pressure is required by the strip to produce plastic deformation because of the increase in back tension. The reduction ratio also increases with the speed ratio at a given back tension as a consequence of an increased $q_{C}$. This finding is consistent with the research result of Sun et al. [7]. The increase rate of the reduction ratio ($\varphi_{r}$) increases with the speed ratio for a given back tension and decreases with back tension for a given speed ratio (Figure 13b). A greater back tension leads to a higher reduction ratio but a lower increase rate of the reduction ratio.

A reduction in the front tension acting on the plastic deformation region because of the elastic deformation of the strip results in a decrease in the reduction ratio (Figure 13a).

**Figure 14:**
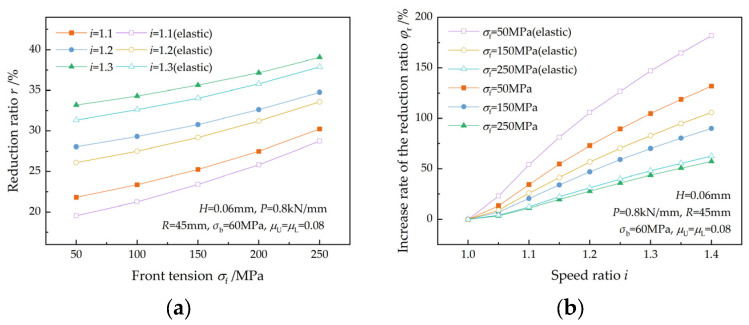
Effects of front tension on (**a**) the reduction ratio and (**b**) the increase rate of the reduction ratio at different speed ratios.

The effect of front tension on the reduction ratio is illustrated in Figure 14, which is similar to that of back tension. The reduction ratio increases with both front tension and the speed ratio and increases faster with the speed ratio at lower front tension. This is in accordance with the findings of Sun et al. [7]. The relative change in front tension caused by the elastic deformation of the strip is greater at a lower front tension, resulting in a greater decrease in the reduction ratio (Figure 14a).

**Figure 15:**
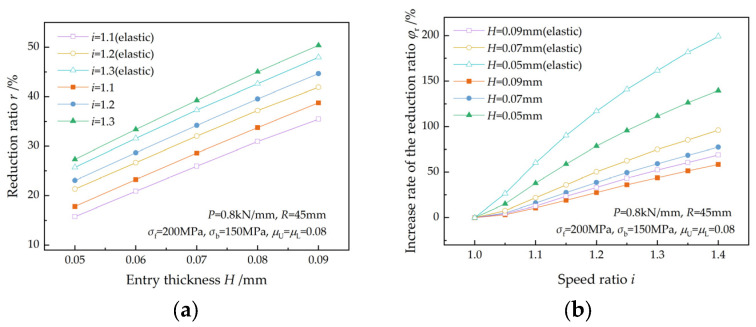
Effects of entry thickness on (**a**) the reduction ratio and (**b**) the increase rate of the reduction ratio at different speed ratios.

The variations in the reduction ratio and the increase rate of the reduction ratio with entry thickness are illustrated in Figure 15. The reduction ratio increases with entry thickness at a given speed ratio and increases with the speed ratio for a given entry thickness. Sun et al. [7] reported a similar finding for thicker strips. For the same entry thickness, the increase rate of the reduction ratio increases with the speed ratio. For the same speed ratio, a higher increase rate can be obtained when the entry thickness is thinner. This indicates that a higher speed ratio is beneficial for ultra-thin strip rolling. The reduction ratio is reduced by the elastic deformation of the strip.

**Figure 16:**
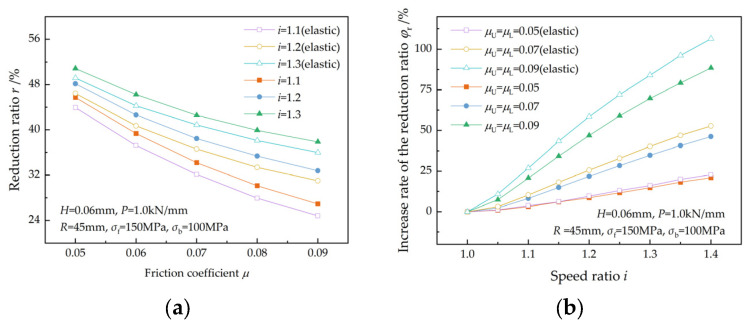
Effects of the friction coefficient on (**a**) the reduction ratio and (**b**) the increase rate of the reduction ratio at different speed ratios.

Figure 16 illustrates the effect of the friction coefficient on the reduction ratio. A decrease in the friction coefficient and an increase in the speed ratio both lead to an increase in the reduction ratio (Figure 16a). This is in accordance with the findings of Zhang et al. [3], Hwang et al. [5], and Sun et al. [7]. The reduction ratio increases more rapidly with the speed ratio at higher friction coefficients because of a higher $q_{C}$ (Figure 16b), which indicates that a higher friction coefficient is a favorable condition for asymmetrical rolling. The elastic deformation of the strip results in a decrease in front tension, consequently leading to a decrease in the reduction ratio.

**Figure 17:**
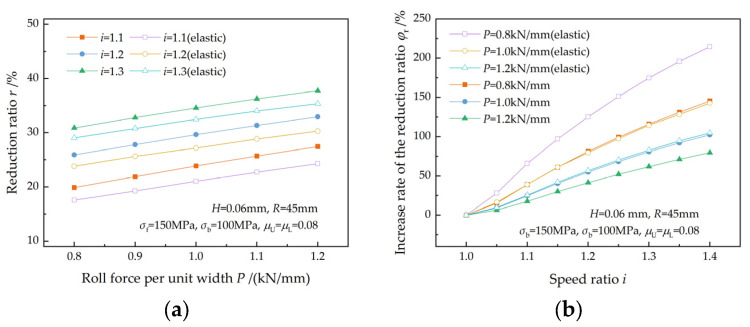
Effects of roll force on (**a**) the reduction ratio and (**b**) the increase rate of the reduction ratio at different speed ratios.

Figure 17 shows the effect of roll force on the reduction ratio. The reduction ratio increases with the roll force (Figure 17a), which is in agreement with Salimi et al. [2], Hwang et al. [5], and Tian et al. [8]. The reduction ratio increases faster at a lower roll force as the speed ratio increases because of a higher increase rate of $q_{C}$ (Figure 17b). This is in agreement with the research result of Sun et al. [7]. The elastic deformation of the strip leads to a lower reduction ratio but a higher increase rate of the reduction ratio.

### 4.3. Verification of Models

The validity of the proposed models was verified by comparing the analytical roll force with the measured experimental values.

**Figure 18:**
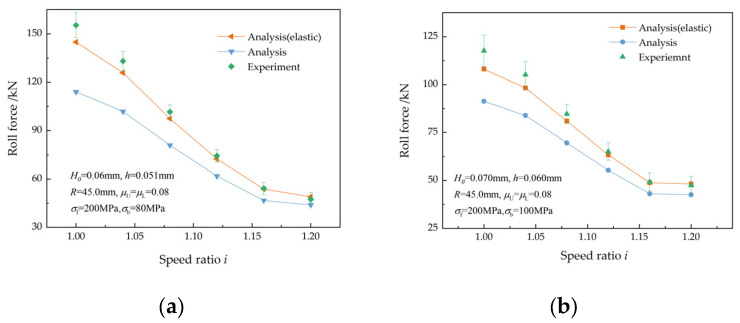
Comparisons between analytical and experimental roll forces for two thicknesses: (**a**) *H* = 0.06 mm and (**b**) *H* = 0.07 mm.

Figure 18 shows a comparison of the experimental roll forces with the analytical values for two ultra-thin strips. The 0.06 mm thick strips were rolled to 0.051 mm with different speed ratios. The calculated and measured roll forces are shown in Figure 18a. As the speed ratio increases, both the analytical and experimental roll forces decrease. This has been reported in references [2,3,7]. A maximum error of 7.2% is found between the experimental and analytical roll forces when the elastic deformation of the strip is taken into account, but the maximum error surges to 36.2% when the elastic deformation of the strip is ignored. The 0.07 mm thick strips were rolled to 0.06 mm in another rolling experiment, and Figure 18b shows the calculated and measured roll forces. The maximum error between the experimental and analytical roll forces is 8.9% when the elastic deformation of the strip is considered, but the maximum error is 28.7% when the elastic deformation of the strip is ignored.

**Figure 19:**
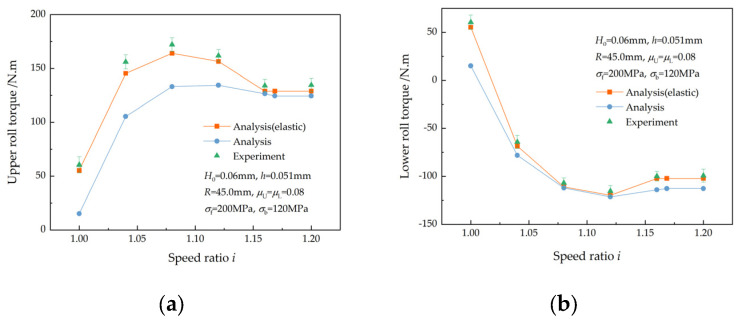
Comparisons of roll torques between the analytical and experimental results: (**a**) the upper roll torque and (**b**) the lower roll torque.

Figure 19a shows a comparison between the analytical values and experimental results of the upper roll torques. When $i<i_{\mathrm{cF}}$, both the analytical and the experimental upper roll torques first increase and then decrease as the speed ratio increases. This is in accordance with the research findings of Salimi et al. [2], Zhang et al. [3], and Sun et al. [7]. When $i>i_{\mathrm{cF}}$, the analytical upper roll torques remain constant, while the experimental upper roll torque increases slightly with the increase in the speed ratio. As the speed ratio increases within the critical speed ratio, $q_{C}$ increases, while the average roll pressure decreases. An increase in $q_{C}$ results in an increase in the upper roll torque, while a decrease in the average roll pressure leads to a decrease in the upper roll torque. When the effect of an increase in $q_{C}$ is greater than the decrease in average roll pressure, the upper roll torque increases; otherwise, the upper roll torque decreases. When $i>i_{\mathrm{cF}}$, both $q_{C}$ and the average roll pressure become constant. The experimental upper roll torque increases slightly because of the increase in the resisting torque in the drive system, which is a consequence of the increased upper roll speed. The maximum error between the analytical and experimental upper roll torque is 9.4% when the elastic deformation of the strip is considered, but the maximum error surges to 75.3% when the elastic deformation of the strip is ignored. The analytical lower roll torques were compared with the experimental results, as illustrated in Figure 19b. As the speed ratio increases within the critical speed ratio, the analytical and experimental lower roll torques initially decrease and then increase. When the speed ratio exceeds the critical speed ratio, the analytical and experimental lower roll torques remain unchanged. The maximum error between the analytical and experimental lower roll torques is 8.5% or 75.0%, respectively, depending on whether the elastic deformation of the strip is considered. The maximum error in both the upper and lower roll torques was observed at a speed ratio of 1.0.

The proposed model is suitable for ultra-thin strips, but it may potentially result in an increase in the error for thick strips because of the simplification.

## 5. Conclusions

A series of mathematical models considering the elastic deformation of the strip were built for the asymmetrical rolling of ultra-thin strips. The models were used to simulate the rolling of ultra-thin strips. The percentages of the three zones and the reduction ratio were analyzed based on the simulation results. The accuracy of the proposed models was verified by experimental rolling. The following conclusions were reached:

- The proposed models are of good accuracy, with the maximum error between the analytical and experimental values for roll torque and roll force being 9.4% and 8.9%, respectively.
- An increase in the speed ratio results in an increase in the percentage of the cross-shear zone. This increase is more pronounced at lower front and back tensions, a lower roll force, a higher friction coefficient, and a thicker entry thickness.
- The elastic deformation of the strip results in an increase in the percentage of the cross-shear zone and a decrease in the percentage of the forward-slip zone, but it only has a slight effect on the percentage of the backward-slip zone.
- An increase in the speed ratio results in an increase in the reduction ratio. This increase is more significantly observed at lower front and back tensions, a higher friction coefficient, and a thinner entry thickness. This indicates that asymmetrical rolling is suitable for ultra-thin strip rolling. An increase in the front tension, the back tension, or the roll force will also result in an increase in the reduction ratio.
- The elastic deformation of the strip leads to a reduced reduction ratio.
- The elastic deformation of the strip yields an effect of reducing tension and the roll pressure.

## Data Availability

Data will be made available on request.

## Nomenclature

B, C, F	backward-slip zone, cross-shear zone, forward-slip zone
Ec, Er	elastic compression zone, elastic recovery zone
B+C+F, B+C, C+F	states of the deformation region
N_U_, N_L_	upper and lower neutral points
*x*, d*x*, ∆x	distance to the exit plane of the plastic deformation region, slab width in the plastic deformation region, slab width in the elastic zone
*H*, *h*, $h_{x}$, $\bar{h}$	entry thickness, exit thickness, thickness at location *x,* average thickness in the plastic deformation region
*h*_U_, *h*_L_	thickness at the upper and lower neutral points
*H*_p_, *h*_p_	thickness at the entry and exit of the plastic deformation region
∆h	thickness reduction
*H*_0_	original thickness of the strip
$\bar{\varepsilon }$	average strain
D_1_, D_2_, D_3_, D_4_ D_5_, D_6_	points on the contact arc
$v_{U}$, $v_{L}$	upper peripheral velocity, lower peripheral velocity
*i*	speed ratio
$i_{\mathrm{cF}}$, $i_{c\;B}$	critical speed ratio for the disappearance of the forward-slip zone and the disappearance of the backward-slip zone
$v_{H}$, $v_{h}$	entry velocity and exit velocity of the strip
$\tau_{U}$, $\tau_{L}$	upper friction stress, lower friction stress
$\mu_{U}$, $\mu_{L}$	upper friction coefficient, lower friction coefficient
$\sigma_{b}$, $\sigma_{f}$	back tension, front tension
$p_{x}$, $\sigma_{x}$	normal and horizonal stresses at location *x*
*K*	plane deformation resistance of the strip
*R*	radius of the work roll
*E,* *ν*	elastic modulus of the work roll, *E* = 206,000 MPa; Poisson ratio of the work roll, ν = 0.3
*θ*, *α*	rolling angle, bite angle
*C*_B_, *C*_C_, *C*_F_	integral constants
$\gamma_{1}$, $\gamma_{2}$	upper neutral angle, lower neutral angle
*l*, *l*_B_, *l*_C_, *l*_F_,	projected contact arc lengths of the plastic deformation region, the backward-slip zone, the cross-shear zone, and the forward-slip zone
*l*_en_, *l*_ex_	projected contact arc lengths of the entry elastic compression zone and the exit elastic recovery zone
$q_{B}$,$q_{C}$,$q_{F}$	percentages of the backward-slip zone, cross-shear zone, and forward-slip zone
$p_{B}$, $p_{C}$, $p_{F}$	roll pressure in the backward-slip zone, cross-shear zone, and forward-slip zone
$p_{n}$, $\sigma_{n}$	normal and horizonal stresses at point n in the elastic zone
$\delta_{1}$, $\delta_{2}$, ζ, λ	variable substitution
$P_{\left(B+C+F\right)}$, $P_{\left(B+C\right)}$, $P_{\left(C+F\right)}$	roll force per unit width in the backward-slip zone, cross-shear zone, and forward-slip zone
$T_{U}$, $T_{L}$	upper roll torque per unit width, lower roll torque per unit width
$\varphi_{r}$	increase rate of the reduction ratio
$r_{0}$, $r_{i}$	reduction ratio at speed ratio 1.0, *i*
